# Development of a Scoring System to Differentiate Severe Fever with Thrombocytopenia Syndrome from Scrub Typhus

**DOI:** 10.3390/v14051093

**Published:** 2022-05-19

**Authors:** Hyoung Sul, Na Ra Yun, Dong-Min Kim, Young Keun Kim, Jieun Kim, Jian Hur, Sook In Jung, Seong Yeol Ryu, Ji Yeon Lee, Kyungmin Huh, Yee Gyung Kwak, Hye Won Jeong, Jung Yeon Heo, Dong Sik Jung, Sun Hee Lee, Sun Hee Park, Joon-Sup Yeom, Hyungdon Lee

**Affiliations:** 1 Department of Infectious Diseases, College of Medicine, Chosun University, Gwangju 61452, Korea; kspt87@naver.com (H.S.); shine@chosun.ac.kr (N.R.Y.); 2 Department of Internal Medicine, Wonju College of Medicine, Yonsei University, Wonju 26426, Korea; amoxj@yonsei.ac.kr; 3 Department of Internal Medicine, College of Medicine, Hanyang University, Seoul 04763, Korea; quidam76@hanyang.ac.kr; 4 Department of Internal Medicine, Yeungnam University Medical Center, Daegu 38541, Korea; sarang7529@yu.ac.kr; 5 Department of Internal Medicine, Chonnam National University Medical School, Gwangju 61469, Korea; sijung@chonnam.ac.kr; 6 Department of Internal Medicine, Keimyung University Dongsan Medical Center, Daegu 42601, Korea; 121rsy@dsmc.or.kr (S.Y.R.); jirong84@dsmc.or.kr (J.Y.L.); 7 Division of Infectious Diseases, Department of Medicine, Samsung Medical Center, Sungkyunkwan University School of Medicine, Seoul 06351, Korea; kyungminhuh.id@gmail.com; 8 Department of Internal Medicine, Inje University Ilsan Paik Hospital, Goyang 10380, Korea; ygkwak@paik.ac.kr; 9 Department of Internal Medicine, College of Medicine, Chungbuk University Hospital, Cheongju 28644, Korea; hwjeong@chungbuk.ac.kr; 10 Department of Infectious Diseases, School of Medicine, Ajou University Hospital, Suwon 16499, Korea; jyeon78@naver.com; 11 Department of Internal Medicine, College of Medicine, Dong-A University Hospital, Busan 49315, Korea; dsjung@dau.ac.kr; 12 Department of Internal Medicine, College of Medicine, Pusan National University Hospital, Busan 50612, Korea; zzanmery@gmail.com; 13 Division of Infectious Diseases, Department of Internal Medicine, College of Medicine, The Catholic University of Korea, Seoul 06591, Korea; sh.park@catholic.ac.kr; 14 Department of Internal Medicine, College of Medicine, Yonsei University, Seoul 03722, Korea; joonsup.yeom@yuhs.ac; 15 Department of Internal Medicine, College of Medicine, Hallym University, Chuncheon 24252, Korea; easydr@hallym.or.kr

**Keywords:** severe fever with thrombocytopenia syndrome, scrub typhus, tsutsugamushi, differential diagnosis, scoring system

## Abstract

Severe fever with thrombocytopenia syndrome (SFTS) and scrub typhus are disorders with similar clinical features; therefore, differentiating between them is difficult. We retrospectively collected data from 183 SFTS and 178 scrub typhus patients and validated an existing scoring system to develop a more sensitive, specific, and objective scoring system. We first applied the scoring systems proposed by Kim et al. to differentiate SFTS from scrub typhus. Multivariable logistic regression revealed that altered mental status, leukopenia, prolonged activated partial thromboplastin time (aPTT), and normal C-reactive protein (CRP) level (≤1.0 mg/dL) were significantly associated with SFTS. We changed the normal CRP level from ≤1.0 mg/dL to ≤3.0 mg/dL and replaced altered mental status with the creatine kinase (CK) level. The modified scoring system showed 97% sensitivity and 96% specificity for SFTS (area under the curve (AUC): 0.983) and a higher accuracy than the original scoring system (*p* = 0.0308). This study’s scoring system had 97% sensitivity and 98% specificity for SFTS (AUC: 0.992) and a higher accuracy than Kim et al.’s original scoring system (*p* = 0.0308). Our scoring system that incorporated leukopenia, prolonged aPTT, normal CRP level (≤3.0 mg/dL), and elevated CK level (>1000 IU/L) easily differentiated SFTS from scrub typhus in an endemic area.

## 1. Introduction

In East Asia, endemic zoonoses such as severe fever with thrombocytopenia syndrome (SFTS) and scrub typhus are becoming major public health concerns [1,2]. SFTS is a novel phlebovirus belonging to the family Bunyaviridae, transmitted by the tick *Haemaphysalis longicornis* [1]. Since its first report in Korea in May 2013, SFTS has affected hundreds of individuals annually (223 cases in 2019) [3], with a mortality rate of approximately 30% [4]. Scrub typhus is caused by intracellular bacteria, *Orientia tsutsugamushi* (*O. tsutsugamushi*)*,* in the family Rickettsiaceae [2]. Yearly, about 10,000 patients are diagnosed with scrub typhus, with a mortality rate of 0.2% in Korea [3].

SFTS and scrub typhus have similar clinical features, which makes it difficult to differentiate between the two. Both diseases are caused by bites from infected ticks or mites in individuals performing outdoor activities in fields, farms, and mountains. Patients commonly have nonspecific symptoms such as fever, headache, fatigue, myalgia, and gastrointestinal symptoms.

As there is no known treatment for SFTS, it is associated with high mortality. However, scrub typhus is treatable with doxycycline or azithromycin, and thus has low mortality. Therefore, differentiating between these two diseases in a clinical setting is essential before confirming laboratory results (reverse transcription-polymerase chain reaction (RT-PCR) findings, >4-fold increases in immunoglobulin (Ig) M or G titers, and culture findings). In this context, Kim et al. developed a scoring system using four parameters: altered mental status, leukopenia, prolonged activated partial thromboplastin time (aPTT), and normal C-reactive protein (CRP) level [5]. However, the statistical power used in their study was low since the number of cases was low (21 SFTS cases and 91 scrub typhus cases). To overcome these limitations, we analyzed a larger number of cases to develop a more sensitive, specific, and objective scoring system than those described in the literature, and validated this scoring system in a larger set of patients [6].

## 2. Methods

### 2.1. Study Details

We retrospectively collected data on 183 SFTS patients and 178 scrub typhus patients who visited 21 study hospitals in South Korea between October 2013 and November 2017. The study protocol was approved by the Institution Review Board of each institution.

To differentiate SFTS from scrub typhus, we first applied scoring systems proposed in other studies (Model A) [5]. Further, to improve the scoring sensitivity, the numerical values were adjusted, and the modified scoring system was applied (Model B). Additionally, a scoring model (Model D) proposed by Park et al. was tested to validate our scoring system [7].

The following models were applied in this study:

Model A—Kim et al.’s original model (altered mental status, leukopenia, prolonged aPTT, normal CRP level [≤1.0 mg/dL]).

Model B—Kim et al.’s modified model (altered mental status, leukopenia, prolonged aPTT, normal CRP level [≤3.0 mg/dL]).

Model C—Our study model (leukopenia, prolonged aPTT, normal CRP level [≤3.0 mg/dL], elevated creatine kinase (CK) level [>1000 IU/L]).

Model D—Park et al.’s model proposed in 2019 (leukopenia, thrombocytopenia, low CRP level [<1.0 mg/dL]) for the prediction of SFTS.

**Figure 1:**
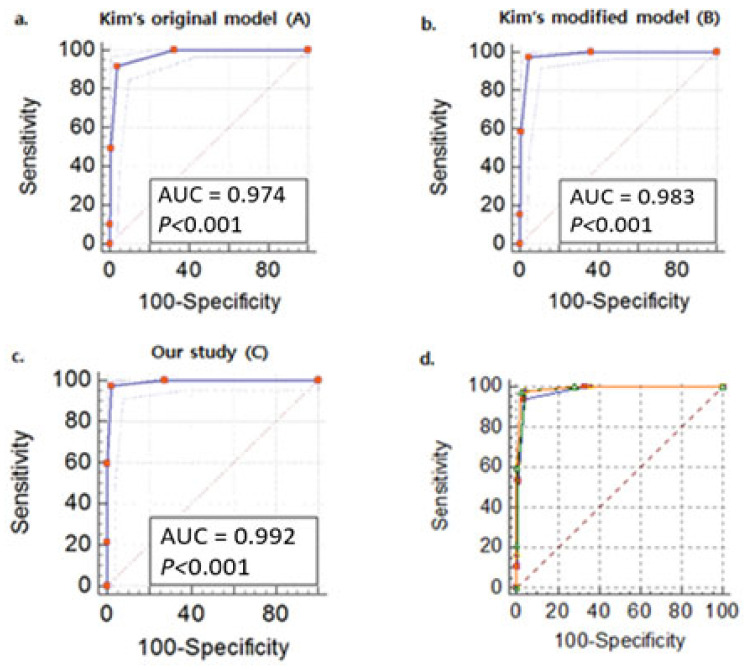
Receiver operating characteristic (ROC) curves of the multivariable logistic regression models (**a**). Model A: altered mental status, leukopenia, prolonged activated partial thromboplastin time (aPTT), normal C-reactive protein (CRP) level (≤1.0 mg/dL), (**b**). Model B: altered mental status, leukopenia, prolonged aPTT, normal CRP level (≤3.0 mg/dL), (**c**). Model C: leukopenia, prolonged aPTT, normal CRP level (≤3.0 mg/dL), elevated creatine kinase level (>1000 IU/L), (**d**) square: model A, circle: Model B, triangle: Model C for the severe fever with thrombocytopenia syndrome predictive model.

Multiple regression analysis was performed based on two previously proposed scoring systems, the modified scoring system and the scoring system developed in our study, to differentiate SFTS from scrub typhus (Figure 1).

### 2.2. Diagnostic Tests

SFTS was diagnosed by detecting SFTS viral RNA from whole blood samples using RT-PCR analysis via DiaStar 2X OneStep RT-PCR Pre-Mix kit (SolGent, Daejeon, Korea) or isolation of the SFTS virus [8]. Scrub typhus was diagnosed by detecting the 56-kDa antigen of *Orientia tsutsugamushi* from 300 µL of whole blood and eschar samples by nested PCR, a ≥4-fold increase in IgM or IgG titer using indirect immunofluorescence assay (IFA), or isolating *O. tsutsugamushi* [9].

### 2.3. Statistical Analysis

Categorical variables were analyzed using Pearson’s chi-square (χ^2^) or Fisher’s exact tests, while continuous variables were analyzed using independent t-tests. Multiple logistic regression analyses were performed to identify the parameters significantly (*p* < 0.05) related to the SFTS differentiation score. The receiver operating characteristic (ROC) curve was constructed for the scoring model. Statistical analyses were performed using IBM SPSS Statistics for Windows, version 22.0 (IBM Corp., Armonk, NY, USA) and MedCalc, version 18.11.6 (MedCalc Software Ltd., Ostend, Belgium).

## 3. Results

The comparative statistical analysis such as Pearson’s chi-square (χ2) or Fisher’s exact tests and independent t-test were performed to clinically differentiate the characteristics and outcomes of SFTS and scrub typhus patients. The clinical characteristics and outcomes of 183 SFTS patients and 178 scrub typhus patients are presented in Supplementary Table S1. *O. tsutsugamushi* 56-kDa nested PCR amplicon sequence analysis identified 154 patients with the Boryong strain, 8 cases with the Karp strain, 4 with the Taguchi strain, and 1 each with the Nishino and Kanda strains. Generally, scrub typhus occurred more often in the autumn and winter months than SFTS (100% vs. 44%, *p* ≤ 0.001). Compared to scrub typhus patients, the time from symptom onset to hospitalization was shorter in SFTS patients (52% vs. 38%, *p* = 0.010; 4.5 ± 3.3 days vs. 7.2 ± 4.0 days, *p* ≤ 0.001). Most SFTS patients in the present study were rural dwellers (93% vs. 69%, *p* ≤ 0.001; 96% vs. 88%, *p* = 0.010) and male (52% vs. 38%, *p* = 0.010) compared to scrub typhus patients; this is significantly different from the study population in Kim et al.’s study.

Supplementary Table S1 represents a detailed multivariate regression analysis of the clinical symptoms, which showed higher incidences of diarrhea (*p* < 0.001), hemorrhagic symptoms (*p* = 0.002), and neurological symptoms in SFTS patients than in scrub typhus patients. In contrast, scrub typhus patients experienced chills (*p* < 0.001), fatigue (*p* < 0.001), ophthalmalgia (*p* < 0.001), sore throat (*p* < 0.001), thirst (*p* < 0.001), cough (*p* < 0.001), anorexia (*p* < 0.001), dyspepsia (*p* < 0.001), skin rash (*p* < 0.001), and tick or chigger bite wound (*p* < 0.001) more often than SFTS patients. Leukopenia (white blood cell (WBC) count < 4000/μL; *p* < 0.001), thrombocytopenia (platelet (PLT) count <150 × 10^3^/μL; *p* < 0.001), normal CRP level (≤3.0 mg/dL; *p*< 0.001), prolonged aPTT (>40 s; *p* < 0.001), and elevated CK levels (>1000 IU/L; *p* < 0.001) were more common in SFTS patients, while leukocytosis (WBC count > 10,000/μL; *p* < 0.001) and elevated aspartate aminotransferase (AST) or alanine aminotransferase (ALT) levels (AST or ALT > 40 IU/L; *p* < 0.001 and *p* < 0.988) were more common in scrub typhus patients. SFTS patients were more frequently admitted to the intensive care unit, required a mechanical ventilator, and had a higher mortality rate than scrub typhus patients. Additionally, scrub typhus patients had myalgia and headache more often and higher alkaline phosphatase levels than SFTS patients.

Supplementary Table S2 shows the results of the univariate regression analysis of variables that differed significantly between SFTS and scrub typhus. To differentiate SFTS from scrub typhus, we first performed a multivariable logistic regression analysis based on the parameters proposed by Kim et al. (Model A) [5]. The regression analysis showed that (Table 1) altered mental status (odds ratio (OR) 5.681, *p* = 0.017), leukopenia (WBC count <4000/μL; OR 75.879, *p* ≤ 0.001), prolonged aPTT (>40 s; OR 80.133, *p* ≤ 0.001), and normal CRP level (≤1.0 mg/dL; OR 166.855, *p* ≤ 0.001) were significantly associated with SFTS as compared to scrub typhus. Each variable was given 1 point for a total score of 0–4 points. The optimal cutoff value in the ROC curve analysis was >1. A score >1 had 92% sensitivity and 96% specificity for the diagnosis of SFTS, with an AUC of 0.974 (0.949–0.989).

The variables of Model A were further analyzed after adjusting their numerical values to increase the scoring sensitivity above 95% and named Model B. The normal CRP level was increased from ≤1.0 to ≤3.0 mg/dL. After logistic regression, altered mental status (OR 15.385, *p* = 0.006), leukopenia (OR 92.573, *p* ≤ 0.001), prolonged aPTT (OR 65.010, *p* ≤ 0.001), and normal CRP level (≤3.0. mg/dL; OR 184.937, *p* ≤ 0.001) were significantly associated with SFTS compared to scrub typhus (Model B). The optimal cutoff value for the ROC curve of the modified scoring system was >1. A score > 1 showed 97% sensitivity and 96% specificity for the diagnosis of SFTS, with an AUC of 0.983 (0.960–0.995). The modified scoring system showed significantly higher accuracy than the original scoring system (AUC 0.978 [0.950–0.992] vs. AUC 0.985 [0.960–0.996], *p* = 0.0487 Table 1).

This study identified four new parameters for predicting SFTS considering all the above study models: leukopenia, prolonged aPTT, normal CRP level (≤3.0 mg/dL), and elevated CK level (> 1000 IU/L). After logistic regression, leukopenia (OR 145.404, *p* ≤ 0.001), prolonged aPTT (OR 250.124, *p* ≤ 0.001), normal CRP level (OR 172.021, *p* ≤ 0.001), and elevated CK level (OR 192.616, *p* = 0.001) were significantly associated with SFTS compared to scrub typhus (Model C). A score > 1 (optimal cutoff value for the ROC curve > 1) had 97% sensitivity and 98% specificity for diagnosing SFTS, with an AUC of 0.992 (0.971–0.999). Our study scoring system showed significantly higher accuracy than Kim’s scoring system (AUC 0.978 [0.950–0.992] vs. AUC 0.992 [0.971–0.999], *p* = 0.0308; Table 1, Supplementary Table S3, Figure 1). Additionally, we applied the scoring system proposed by Park et al. With an AUC of 0.945 (0.949–0.989), a score of >1 demonstrated 84% sensitivity and 95% specificity for the diagnosis of SFTS (Supplementary Figure S1 and Table 1).

## 4. Discussion

Cases of SFTS have recently been documented in scrub typhus endemic areas (China, Japan, and Korea), as well as in Vietnam [6,8]. The first SFTS virus and *O. tsutsugamushi* co-infection cases were reported in Korea in 2017 [10]. Patients with clinically suspected rickettsioses, such as spotted fever and scrub typhus, and confirmed SFTS infection were studied retrospectively in a 2017 study in Japan [11]. Therefore, clinical differentiation between SFTS and scrub typhus is critical due to their similarity in symptoms and prevalence in similar areas.

According to a Chinese study on scrub typhus patients, the interval between illness onset and diagnosis decreased from 7 days (2006) to 5 days (2016). Although this is encouraging, the time taken for diagnosis remains significant [12]. Hence, numerous studies have focused on identifying methods that quickly and easily differentiate between these two diseases before serologic or molecular diagnosis results become available.

Park et al. analyzed 107 and 255 patients with SFTS and scrub typhus, respectively [7], to propose an SFTS prediction scoring system considering parameters such as leukopenia (WBC count <4000/mm^3^), thrombocytopenia (PLT count <80,000/mm^3^), and low CRP level (<1 mg/dL). Each parameter was given 1 point for a total score of 0–3 points, and the presence of any one of these symptoms indicated SFTS. The reported optimal cutoff value was ≥2 by ROC curve analysis. As per the study, a score ≥ 2 had 93% sensitivity and 96% specificity for the diagnosis of SFTS, with an AUC of 0.972 (0.952–0.990). We applied the scoring system proposed by Park et al. (Model D) in our study. A score of >1 had 84% sensitivity and 95% specificity for the diagnosis of SFTS, with an AUC of 0.945 (0.949–0.989) (Supplementary Figure S1 and Table 1). The sensitivity data calculated was 84%, lower than that reported by Park et al. (93%).

Further, Kim et al. reported that their SFTS prediction scoring system, composed of four parameters, had 100% sensitivity and 97% specificity [5]. However, when we applied the scoring system proposed by Kim et al., we achieved a sensitivity of just 92%. Since altered mental status can be subjective, we tried to identify variables that could be objectively evaluated. Hence, altered mental status was excluded, and CK was added to our scoring system, which showed significantly higher accuracy than that of the Park’s scoring system (AUC 0.945 [0.908–0.970] vs. 0.992 [0.971–0.999], *p* = 0.0006). These parameters are objective indicators that can be easily tested within a few hours in the emergency room. Therefore, we can easily differentiate SFTS from scrub typhus in endemic areas using a scoring system incorporating leukopenia, aPTT prolongation, normal CRP level, and elevated CK level.

Laboratory testing is required for a specific diagnosis of SFTS and scrub typhus; however, confirmation may take longer. Identifying viral RNA in a patient’s blood via RT-PCR is used to diagnose SFTS [1]. Usually, there is no standardized or commercially available RT-PCR for SFTS, and IFA testing is the current gold standard for diagnosing scrub typhus [13]. However, a single IFA measurement is sometimes insufficient for a definitive diagnosis. As a result, we hypothesized that clinical distinction could be relevant to developing a scoring function for differentiating SFTS from scrub typhus.

Our study has some limitations. We performed a retrospective study by collecting data on patients suspected of SFTS and scrub typhus from 21 university hospitals. Thus, there is a probability of selection bias towards more severe diseases. Additionally, based on the retrospective data, we built our scoring system. As a result, additional prospective investigations are required to confirm our scoring approach on an independent dataset.

We have proposed an SFTS prediction scoring system including leukopenia (WBC count < 4000/μL), aPTT prolongation (>40 s), normal CRP level (≤3.0 mg/dL), and elevated CK level (>1000 IU/L) to differentiate SFTS from scrub typhus. This scoring system was significantly more sensitive than the previously reported scoring systems, making it easier to differentiate SFTS from scrub typhus clinically. The findings of this study point to a simple and effective scoring method for predicting SFTS in patients with endemic zoonoses. This strategic approach is expected to improve clinical outcomes by allowing early differentiation of SFTS from other endemic zoonoses, particularly by healthcare practitioners.

## Figures and Tables

**Table 1:** Multivariable logistic regression analysis parameters predictive of severe fever with thrombocytopenia syndrome and the diagnostic performance of the clinical scoring system in differentiating severe fever with thrombocytopenia syndrome.

Model	Multivariable Logistic Regression Analysis	Odds Ratio (95% CI)	*p-* Value	AUC (95% CI)	*p-* Value	Sensitivity (95% CI)	Specificity (95% CI)
A	Altered mental status	5.681 (1.369–23.571)	0.017	0.974 (0.949–0.989)	<0.001	91.9 (86.3–95.7)	96.3 (91.6–98.8)
	Leukopenia (WBC count <4000/μL)	75.879 (14.418–399.323)	<0.001				
	Prolonged aPTT (>40 s)	80.133 (14.369–446.877)	<0.001				
	Normal CRP level (≤1.0 mg/dL)	166.855 (23.482–1185.613)	<0.001				
B	Altered mental status	15.385 (2.216–106.828)	0.006	0.983 (0.960–0.995)	<0.001	97.3 (93.2–99.3)	95.6 (90.6–98.4)
	Leukopenia (WBC count < 4000/μL)	92.573 (14.971–572.430)	<0.001				
	Prolonged aPTT (>40 s)	65.010 (10.510–402.105)	<0.001				
	Normal CRP level (≤3.0 mg/dL)	184.937 (35.731–957.207)	<0.001				
C	Leukopenia (WBC count < 4000/μL)	145.404 (12.686–1666.604)	<0.001	0.992 (0.971–0.999)	<0.001	97.3 (92.4–99.4)	97.8 (93.6–99.5)
	Prolonged aPTT (>40 s)	250.124 (18.403–3399.536)	<0.001				
	Normal CRP level (≤3.0 mg/dL)	172.021 (26.289–1125.629)	<0.001				
	Elevated CK level (>1000 IU/L)	192.616 (8.307–4466.445)	0.001				
D	Leukopenia (WBC count <4000/μL)	14.312 (6.228–32.887)	<0.001	0.945 (0.914–0.968)	<0.001	83.7 (77.2–89.0)	95.0 (90.0–98.0)
	Thrombocytopenia (PLT < 80 k)	7.231 (3.091–16.912)	<0.001				
	Normal CRP level (<1.0 mg/dL)	73.889 (15.752–346.587)	<0.001				

Abbreviations: CI, confidence interval; WBC, white blood cell; aPTT, activated partial thromboplastin time; CRP, C-reactive protein; CK, creatine kinase; PLT, platelet count test.

## Data Availability

We shared our data to figshare with doi https://doi.org/10.6084/m9.figshare.16737928 (accessed on 1 March 2022).

## Supplementary Materials

The following supporting information can be downloaded at: https://www.mdpi.com/article/10.3390/v14051093/s1, Figure S1: Receiver operating characteristic (ROC) curves of the multivariable logistic regression models (a. model D: leukopenia, thrombocytopenia, normal CRP level (<1.0 mg/dL), b. square: model A (altered mental status, leukopenia, prolonged aPTT, normal CRP level [≤1.0 mg/dL]), circle: model B (altered mental status, leukopenia, prolonged aPTT, normal CRP level [≤3.0 mg/dL]), triangle: model C (leukopenia, prolonged aPTT, normal CRP level [≤3.0 mg/dL], elevated CK level [>1000 IU/L]), dotted line: model D (leukopenia, thrombocytopenia, normal CRP level [<1.0 mg/dL]) for the SFTS predictive model. Table S1: Clinical characteristics and outcomes of severe fever with thrombocytopenia syndrome and scrub typhus. Table S2: Univariable logistic regression analysis of parameters predictive of severe fever with thrombocytopenia syndrome. Table S3: Diagnostic performance of the clinical scoring system in differentiating severe fever with thrombocytopenia syndrome
