# Supplementary material for: Development of a Scoring System to Differentiate Severe Fever with Thrombocytopenia Syndrome from Scrub Typhus
Source: Viruses. 2022 May 19;14(5):1093. doi: 10.3390/v14051093 (PMC9143636; doi:10.3390/v14051093)
Supplement: Supplementary file 1 [file viruses-14-01093-s001.zip › Figure S1.pdf]

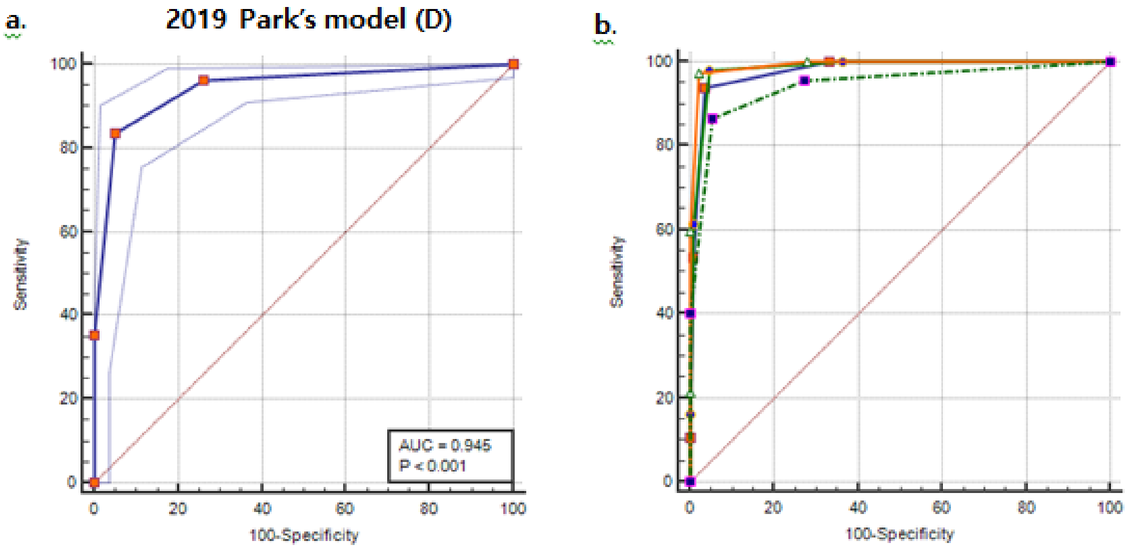

Figure S1. Receiver operating characteristic (ROC) curves of the multivariable logistic regression models (**a.** model D: leukopenia, thrombocytopenia, normal CRP level (<1.0 mg/dL), **b.** square: model A (altered mental status, leukopenia, prolonged aPTT, normal CRP level [≤1.0 mg/dL]), circle: model B (altered mental status, leukopenia, prolonged aPTT, normal CRP level [≤3.0 mg/dL]), triangle: model C (leukopenia, prolonged aPTT, normal CRP level [≤3.0 mg/dL], elevated CK level [>1000 IU/L]), dotted line: model D (leukopenia, thrombocytopenia, normal CRP level [<1.0 mg/dL]) for the SFTS predictive model> .
