# Supplementary material for: Development of a Scoring System to Differentiate Severe Fever with Thrombocytopenia Syndrome from Scrub Typhus
Source: Viruses. 2022 May 19;14(5):1093. doi: 10.3390/v14051093 (PMC9143636; doi:10.3390/v14051093)
Supplement: Supplementary file 1 [file viruses-14-01093-s001.zip › Table S1.pdf]

**Table S1.** Clinical characteristics and outcomes of severe fever with thrombocytopenia syndrome and scrub typhus.

| Variable | SFTS ($n$=183) No. (%) or Mean (SD) | Scrub typhus ($n$=178) No. (%) or Mean (SD) | $p$ value |
|---|---|---|---|
| Season (months) | | | <.001 |
| Spring–Summer (March–August) | 102 (56) | 0 | |
| Autumn–Winter (September–February) | 81 (44) | 178 (100) | |
| Geographic distribution (residential area) | | | <.001 |
| Metropolitan area | 12 (7) | 55 (31) | |
| Province | 171 (93) | 121 (69) | |
| Geographic distribution (infected area) | | | .010 |
| Metropolitan area | 8 (4) | 16 (12) | |
| Province | 172 (96) | 113 (88) | |
| Age, mean (SD), year | 66 (14) | 69 (13) | .044 |
| Male sex | 95 (52) | 68 (38) | .010 |
| Underlying disease | | | |
| Previously healthy | 85 (46) | 67 (39) | .141 |
| Diabetes | 37 (20) | 39 (23) | .477 |
| Hypertension | 71 (39) | 62 (37) | .715 |
| Cerebrovascular disease | 12 (7) | 16 (10) | .313 |
| Congestive heart failure | 3 (2) | 6 (4) | .321 |
| Asthma | 1 (1) | 0 (0) | >.99 |
| COPD | 3 (2) | 3 (2) | >.99 |
| Solid tumor | 5 (3) | 8 (5) | .304 |
| Chronic liver disease | 8 (5) | 5 (3) | .366 |
| Chronic kidney disease | 3 (2) | 3 (2) | >.99 |
| Immunosuppressive condition | 0 | 0 | |
| Symptom duration before hospital visit, mean (SD), day | 4.5 (3.3) | 7.2 (4.0) | <.001 |
| Clinical characteristics | | | |
| Fever | 169 (93) | 161 (93) | .905 |
| Chillness | 115 (65) | 143 (90) | <.001 |
| Myalgia | 86 (47) | 121 (74) | <.001 |
| Fatigue | 48 (27) | 133 (91) | <.001 |
| Ophthalmalgia | 1 (1) | 37 (26) | <.001 |
| Sore throat | 7 (4) | 44 (32) | <.001 |
| Thirst | 15 (9) | 111 (75) | <.001 |
| Cough | 26 (15) | 50 (33) | <.001 |
| Dyspnea | 34 (20) | 32 (22) | .557 |
| GI symptoms | | | |
| Anorexia | 67 (38) | 105 (71) | <.001 |
| Nausea | 68 (39) | 66 (40) | .762 |
| Vomiting | 34 (19) | 33 (21) | .765 |
| Diarrhea | 60 (34) | 29 (18) | <.001 |
| Dyspepsia | 12 (7) | 49 (34) | <.001 |
| Abdominal pain | 39 (22) | 39 (25) | .514 |
| Hemorrhagic symptoms | 28 (15) | 9 (5) | .002 |
| Hemoptysis | 4 (2) | 4(3) | .735 |
| Hematemesis/melena | 5 (3) | 3 (2) | >.99 |
| Purpura | 6 (4) | 3 (2) | .736 |
| CNS symptoms | | | |
| Headache | 55 (31) | 103 (63) | <.001 |
| Neck stiffness | 13 (8) | 13(11) | .265 |
| Altered mental status | 69 (39) | 27 (16) | <.001 |
| Skin rash | 28 (16) | 142 (83) | <.001 |
| Conjunctival injection | 12 (7) | 20 (15) | .026 |

| | | | |
|---|---|---|---|
| Lymphadenopathy | 18 (10) | 24 (18) | .066 |
| Tick or chigger bite wound | 54 (31) | 154 (92) | <.001 |
| Typical eschar | 22 (17) | 154 (100) | <.001 |
| Laboratory findings | | | |
| Leukopenia (WBC count<4000/μL) | 164 (90) | 25 (15) | <.001 |
| Leukocytosis (WBC count>10,000/μL) | 4 (2) | 57 (34) | <.001 |
| WBC count, mean (SD), WBCs/μL | 2457 (2670) | 8454 (4944) | <.001 |
| Thrombocytopenia (PLT count <150X$10^3$/μL) | 173 (95) | 114 (68) | <.001 |
| Thrombocytopenia (PLT count <100X$10^3$/μL) | 137 (75) | 43 (26) | <.001 |
| Thrombocytopenia (PLT count <50X$10^3$/μL) | 53 (29) | 9 (5) | <.001 |
| Platelet count, mean (SD), x$10^3$/μL | 77 (43) | 185 (577) | .016 |
| Anemia (Hb<11g/dL) | 14 (8) | 23 (14) | .066 |
| Hemoglobin, mean (SD), g/dL | 13.5 (1.8) | 13.8 (11.4) | .800 |
| Prolonged PT (INR >1.3) | 8 (5) | 10 (6) | .750 |
| INR, mean (SD) | 1.1 (0.2) | 2.0 (8.1) | .164 |
| Prolonged aPTT (>40 seconds) | 87 (55) | 6 (4) | <.001 |
| aPTT, mean (SD), seconds | 49 (44) | 33 (14) | <.001 |
| Normal CRP level (≤3.0 mg/dL) | 146 (88) | 9 (6) | <.001 |
| Normal CRP level (≤1.0 mg/dL) | 110 (66) | 2 (1) | <.001 |
| CRP, mean (SD), mg/dL | 2.0 (6.9) | 11.2 (9.5) | <.001 |
| Renal dysfunction (Cr>1.30) | 33 (22) | 34 (27) | .292 |
| Creatinine, mean (SD), mg/dL | 1.2 (1.0) | 1.7 (5.9) | .309 |
| Abnormal LFT (AST or ALT>40 IU/L) | 141 (78) | 154 (92) | <.001 |
| AST, mean (SD), IU/L | 226 (418) | 118 (111) | .001 |
| ALT, mean (SD), IU/L | 87 (119) | 87 (80) | .988 |
| Alkaline phosphatase, mean (SD), IU/L | 90 (94) | 125 (106) | .007 |
| Total bilirubin, mean (SD), mg/dL | 0.6 (0.6) | 0.9 (0.5) | <.001 |
| Rhabdomyolysis feature (CK>1000IU/L) | 58 (45) | 5 (3) | <.001 |
| Creatine kinase, mean (SD), IU/L | 1866 (4747) | 227 (558) | <.001 |
| Lactate dehydrogenase, mean (SD), IU/L | 1086 (1825) | 849 (258) | .144 |
| Complications | | | |
| Meningoencephalitis | 12 (7) | 21 (13) | .056 |
| Seizure | 9 (5) | 0 | .004 |
| Arrhythmia | 10 (6) | 3 (2) | .070 |
| Pneumonia | 5 (3) | 15 (9) | .011 |
| Hemophagocytic lymphohistiocytosis | 4 (2) | 0 | .143 |
| Clinical course | | | |
| ICU admission | 73 (40) | 15 (9) | <.001 |
| Mechanical ventilation | 32 (23) | 7 (4) | <.001 |
| In-hospital death | 40 (22) | 4 (2) | <.001 |

Abbreviations: COPD, Chronic obstructive pulmonary disease; GI, gastrointestinal; CNS, central nervous system; ICU, intensive care unit
