# Supplementary material for: Development of a Scoring System to Differentiate Severe Fever with Thrombocytopenia Syndrome from Scrub Typhus
Source: Viruses. 2022 May 19;14(5):1093. doi: 10.3390/v14051093 (PMC9143636; doi:10.3390/v14051093)
Supplement: Supplementary file 1 [file viruses-14-01093-s001.zip › Table S2.pdf]

Table S2. Univariable logistic regression analysis of parameters predictive of severe fever with thrombocytopenia syndrome.

Univariable Logistic Regression Analysis	Odds Ratio (95% CI)	*p* Value
Season	0.00	0.996
Geographic distribution (residential area)	6.477 (3.326–12.613)	<0.001
Geographic distribution (infected area)	3.044 (1.261–7.349)	0.013
Age	0.984 (0.968–1.000)	0.046
Male sex	0.578 (0.380–0.879)	0.010
Chillness	0.204(0.112–0.373)	<0.001
Myalgia	0.311(0.197–0.491)	<0.001
Fatigue	0.039 (0.020–0.073)	<0.001
Ophthalmalgia	0.021 (0.003–0.159)	<0.001
Sore throat	0.093 (0.040–0.214)	<0.001
Thirst	0.032 (0.017–0.062)	<0.001
Cough	0356 (0.208–0.610)	<0.001
Anorexia	0.258 (0.162–0.410)	<0.001
Diarrhea	2.447 (1.472–4.067)	0.001
Dyspepsia	0.147 (0.075–0.291)	<0.001
Hemorrhagic symptoms	3.212 (1.468–7.028)	0.003
Headache	0.260 (0.166–0.408)	<0.001
Altered mental status	3.376 (2.028-5.621)	<0.001
Skin rash	0.040 (0.023–0.070)	<0.001
Conjunctival injection	0.431 (0.203–0.917)	0.029
Tick or chigger bite wound	0.040 (0.021–0.075)	<0.001
Leukopenia (WBC count<4000/μL)	52.116 (27.315–99.434)	<0.001
Leukocytosis (WBC count>10,000/μL)	0.044 (0.015–0.124)	<0.001
Thrombocytopenia (PLT count <150x10^3/μL)	8.195 (4.009–16.751)	<0.001
Thrombocytopenia (PLT count <100x10^3/μL)	8.658 (5.350–14.009)	<0.001
Thrombocytopenia (PLT count <50x10^3/μL)	7.203 (3.424–15.151)	<0.001
Prolonged aPTT (>40 seconds)	32.729 (13.659–78.420)	<0.001
Normal CRP level (≤3.0 mg/dL)	107.067 (47.101–243.379)	<0.001
Normal CRP level (≤1.0 mg/dL)	136.518 (32.590–571.861)	<0.001
Abnormal LFT (AST or ALT level >40 IU/L)	0.305 (0.156–0.595)	<0.001
Alkaline phosphatase level	0.996 (0.994–0.999)	0.012
Total bilirubin level	0.228 (0.118–0.440)	<0.001
Elevated CK level (>1000 IU/L)	23.200 (8.905–60.441)	<0.001

Abbreviations: CI, confidence interval; WBC, white blood cell; aPTT, activated partial thromboplastin time; CRP, C-reactive protein; LFT, liver function test; AST, aspartate aminotransferase; ALT, alanine aminotransferase; CK, creatine kinase.
