# Supplementary material for: Development of a Scoring System to Differentiate Severe Fever with Thrombocytopenia Syndrome from Scrub Typhus
Source: Viruses. 2022 May 19;14(5):1093. doi: 10.3390/v14051093 (PMC9143636; doi:10.3390/v14051093)
Supplement: Supplementary file 1 [file viruses-14-01093-s001.zip › Table S3.pdf]

Table S3. Diagnostic performance of the clinical scoring system in differentiating severe fever with thrombocytopenia syndrome.

Clinical Score* (*n*=247)	SFTS (*n*=112)	Scrub Typhus (*n*=135)	Sensitivity (95% CI)	Specificity (95% CI)	Positive Likelihood Ratio (95% CI)	Negative Likelihood Ratio (95% CI)	Positive Predictive Value	Negative Predictive Value
>0	112	37	100.0 (96.8–100.0)	72.6 (64.3–79.9)	3.7 (2.8–4.8)	0	75.2 (69.7–79.9)	100.0
>1	109	3	97.3 (92.4–99.4)	97.8 (93.6–99.5)	43.8 (14.3–134.1)	0.03 (0.009–0.08)	97.3 (92.2–99.1)	97.8 (93.5–99.3)
>2	67	0	59.8 (50.1–69.0)	100.0 (97.3–100.0)	NA	0.40 (0.3–0.5)	100.0	75.0 (70.5–79.0)
>3	24	0	21.4 (14.2–30.2)	100.0 (97.3–100.0)	NA	0.79 (0.7–0.9)	100.0	60.5 (58.2–62.8)

* Scoring system = (1 x leukopenia) + (1 x prolonged aPTT) + (1 x normal CRP level ≤3.0 mg/dL) + (1 x elevated CK level). Abbreviations: CI, confidence interval; SFTS, severe fever with thrombocytopenia syndrome; aPTT, activated partial thromboplastin time; CRP, C-reactive protein; CK, creatine kinas
